# Neuroprotective Effect of Carotenoid-Rich *Enteromorpha prolifera* Extract via TrkB/Akt Pathway against Oxidative Stress in Hippocampal Neuronal Cells

**DOI:** 10.3390/md18070372

**Published:** 2020-07-19

**Authors:** Seung Yeon Baek, Mee Ree Kim

**Affiliations:** Department of Food and Nutrition, Chungnam National University, Daejeon 34134, Korea; qor7683@o.cnu.ac.kr

**Keywords:** *Enteromorpha prolifera*, oxidative stress, apoptosis, BDNF, TrkB/Akt pathway

## Abstract

In this study, we found that *E. prolifera* extract (EAEP) exhibits neuroprotective effects in oxidative stress-induced neuronal cells. EAEP improved cell viability as well as attenuated the formation of intracellular reactive oxygen species (ROS) and apoptotic bodies in glutamate-treated hippocampal neuronal cells (HT-22). Furthermore, EAEP improved the expression of brain-derived neurotrophic factor (BDNF) and antioxidant enzymes such as heme oxygenase-1 (HO-1), NAD(P)H quinine oxidoreductase-1 (NQO-1), and glutamate–cysteine ligase catalytic subunit (GCLC) via the tropomyosin-related kinase receptor B/ protein kinase B (TrkB/Akt) signaling pathway. In contrast, the pre-incubation of K252a, a TrkB inhibitor, or MK-2206, an Akt-selective inhibitor, ameliorated the neuroprotective effects of EAEP in oxidative stress-induced neuronal cells. These results suggest that EAEP protects neuronal cells against oxidative stress-induced apoptosis by upregulating the expression of BDNF and antioxidant enzymes via the activation of the TrkB/Akt pathway. In conclusion, such an effect of EAEP, which is rich in carotenoid-derived compounds, may justify its application as a food supplement in the prevention and treatment of neurodegenerative disorders.

## 1. Introduction

In recent decades, the number of patients diagnosed with neurodegenerative diseases, such as Alzheimer’s and Parkinson’s disease, has increased continuously along with the extended lifespan and environmental pollution worldwide [1,2]. The pathophysiology of neurodegenerative diseases is closely related to the generation of reactive oxygen species (ROS), which results in protein and DNA damage, inflammation, tissue damage and subsequently induces apoptosis in neuronal cells [3,4]. Accordingly, one of the key neuroprotective strategies entails regulation of ROS generation in order to prevent or treat neurodegenerative diseases [5]. Another preventive and treatment strategy for neurodegenerative disease involves activating the production of brain-derived neurotrophic factor (BDNF) [6]. BDNF is released from the central nervous system and plays an important role in cell proliferation, protection, synaptic function, morphogenesis, and plasticity mediated via tropomyosin-related kinase receptor B (TrkB) [7,8]. Most studies investigating BDNF have reported that the expression of BDNF protects neuronal cells against oxidative stress and decreases the risk of neurodegenerative disorders in the brain [8,9]. Recent studies suggest that some natural antioxidant compounds suppress the oxidative stress induced by ROS and activate the BDNF/TrkB pathway in neuronal cells [9,10,11].

Marine seaweeds possess biochemical properties that may be exploited to develop therapeutic and functional foods. Marine seaweeds are considered an abundant source of antioxidants including flavonoids, phenolic compounds, carotenoids, and chlorophyll [12,13,14,15]. *Enteromorpha prolifera* (EP) belongs to the phylum Chlorophyta, class Chlorophyceae, order *Ulvales*, and genus *Enteromorpha* [16]. It is cultivated worldwide along the seashore and is used to treat symptoms including epistaxis and signs of inflammation in eastern Asia [17]. Polysaccharides derived from EP have attracted the attention of investigators involved in their identification and determination of structure and function including antioxidant, anti-inflammatory, anti-diabetic, and anti-apoptotic activities [17,18,19,20]. However, EP is a source of polysaccharides, crude fiber and protein, as well as unsaturated fatty acids such as linoleic acid, linolenic acid, EPA, DHA, minerals, and vitamins [21]. Furthermore, EP contains diverse phytochemicals such as chlorophyll, phycocyanin, flavonoids, and phenolic compounds, which affect the antioxidant activity of the marine algal extract [22,23]. The anti-diabetic effect of flavonoids and polyphenols in the EP extract was established in the intestinal microflora of type 2 diabetic mice [24,25]. In addition, in a previous report, we investigated that the antioxidant activity of an ethyl acetate fraction of EP extract (EAEP) was stronger than that of aqueous and 95% ethanol extract [23]. In the report, we found that EAEP is rich in carotenoid-derived dihydroactinidiolide and carotenoids including canthaxanthin, violaxanthin, and fucoxanthin, which exhibit potent neuroprotective activity [23,26,27,28,29]. Despite extensive analyses of the EP extract, few studies have investigated the antioxidant and neuroprotective effects associated with phytochemicals.

In this study, we investigated the neuroprotective role of EAEP against oxidative stress-induced neurodegeneration in hippocampal neuronal cells (HT-22) mediated via TrkB/Akt pathway.

## 2. Results

### 2.1. Neuroprotective Effect of EAEP on Glutamate-Induced Oxidative Stress in HT-22 Cells

**Figure 1:**
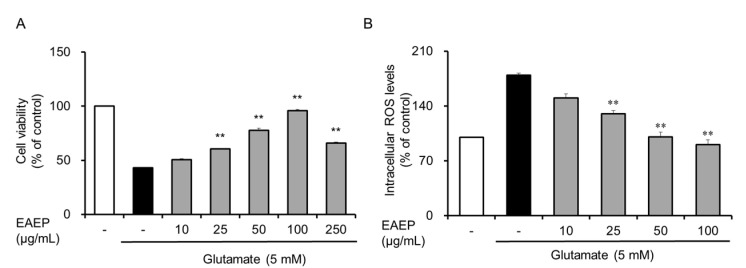
Effect of ethyl acetate extract of *Enteromorpha prolifera* (EAEP) on glutamate-induced cytotoxicity and reduction in reactive oxygen species (ROS) in hippocampal neuronal cells (HT-22). HT-22 cells, seeded on 96-well-plates and incubated for 24 h, were treated with or without EAEP (0–100 µg/mL) for 30 min before glutamate challenge (5 mM). After 12 h, cell viability and intracellular ROS levels were estimated as described in the Materials and Methods. (**A**) Cell viability. (**B**) ROS level. Data represent the mean ± SEM values in triplicate; ** *p* < 0.01 versus glutamate-treated group. − denotes absence of EAEP.

Glutamate is an excitatory neurotransmitter in the central nervous system [30]. High concentrations of glutamate inhibit the uptake of *N*-acetyl cysteine, which results in the reduction in intracellular glutathione levels and subsequent oxidative stress associated with cell death such as apoptosis and necrosis [31]. First, we investigated whether EAEP prevented glutamate-induced oxidative stress in hippocampal neuronal cells because EAEP has been reported to exhibit high antioxidant activity [21,23]. The incubation of HT-22 cells with EAEP (10–100 μg/mL) prior to glutamate treatment improved cell viability up to a concentration of 100 μg/mL (Figure 1A). In particular, exposure to EAEP 100 μg/mL completely restored cell viability to the same level as in the control group. As shown in Figure 1B, glutamate-induced HT-22 cells generated intracellular ROS. In contrast, EAEP dose-dependently reduced the intracellular ROS level (Figure 1B). These results suggest that EAEP attenuated neuronal cell death against oxidative stress by regulating ROS generation. Thus, EAEP may play a neuroprotective role in glutamate-induced HT-22 cells.

### 2.2. Inhibitory Effect of EAEP on Oxidative Stress-Induced Apoptosis in HT-22 Cells

**Figure 2:**
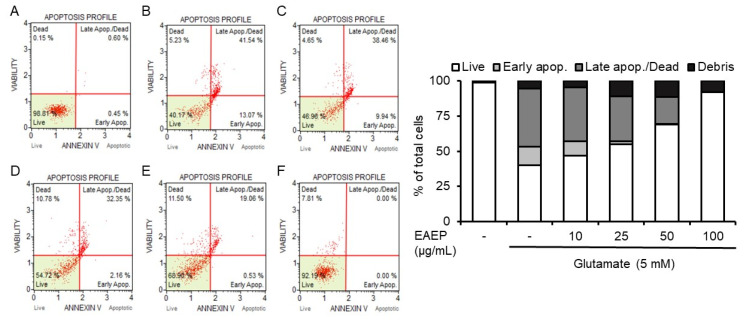
Inhibitory effect of ethyl acetate extract of *Enteromorpha prolifera* (EAEP) on oxidative stress-induced apoptosis in HT-22 cells. HT-22 cells, seeded on 60-mm dishes and incubated for 24 h, were treated with or without EAEP (0–100 µg/mL) for 30 min before glutamate challenge (5 mM). After 12 h, the harvested cells including apoptotic and necrotic cells were analyzed by flow cytometry as described in Section 2. (**A**) vehicle control; (**B**) glutamate alone; (**C**) glutamate + 10 µg/mL EAEP; (**D**) glutamate + 25µg/mL EAEP; (**E**) glutamate + 50 µg/mL EAEP; (**F**) glutamate + 100 µg/mL EAEP. − denotes absence of EAEP.

Next, we investigated inhibitory effect of EAEP against oxidative stress in glutamate-induced HT-22 cells because glutamate induces apoptosis in HT-22 cells [30]. As shown in Figure 2, flow cytometry using Annexin V led to a significant increase in apoptotic bodies in glutamate-treated HT-22 cells. Meanwhile, pretreatment with EAEP dose-dependently reduced the number of apoptotic bodies. Furthermore, treatment with EAEP 100 μg/mL restored the level of apoptotic bodies to that of the control group, indicating that EAEP protected neuronal cells by inhibiting oxidative stress-induced apoptotic cell death.

### 2.3. EAEP Activates the Expression of Antioxidant Enzymes via Akt/Nrf2 Pathway

**Figure 3:**
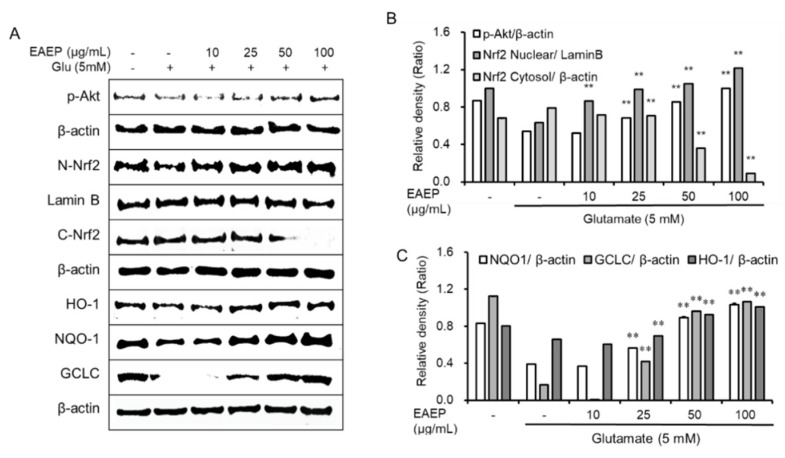
Treatment with ethyl acetate extract of *Enteromorpha prolifera* (EAEP) activates the expression of antioxidant enzymes via Akt/Nrf2 pathway. (**A**) Immunoblotting for protein expression related with Akt/Nrf2 pathway, (**B**) relative density of p-Akt, nuclear and cytosolic Nrf2, (**C**) NQO-1, GLCL, and HO-1. HT-22 cells were seeded on a 60-mm dish, and incubated for 24 h. The cells were challenged with glutamate after pre-incubation with or without EAEP (0–100 µg/mg) for 30 min. After 12 h, the expression of p-Akt, nuclear and cytosolic Nrf2, HO-1, NQO-1, GCLC or β-actin was examined as described in the Materials and Methods. The data were based on three independent experiments. ** *p* < 0.01 versus glutamate-treated group. − denotes absence of EAEP.

We evaluated the effects of EAEP on the expression of antioxidant enzymes to identify the mechanism underlying prevention of oxidative stress-induced apoptosis (Figure 3). It was reported that activation of Akt/Nrf2 pathway influenced the neuroprotective activity [31,32]. Treatment with EAEP led to upregulation of the expression of p-Akt and the nuclear translocation Nrf2. It also boosted the levels of antioxidant enzymes including HO-1, glutamate–cysteine ligase catalytic subunit (GCLC), and NQO-1 in glutamate-treated HT-22 cells. Based on these results, the antioxidant action of EAEP seems to be mediated via Akt/Nrf2 signaling pathway.

### 2.4. Effect of Activated EAEP on the Expression of BDNF Mediated via ERK/CREB/TrkB Pathway

**Figure 4:**
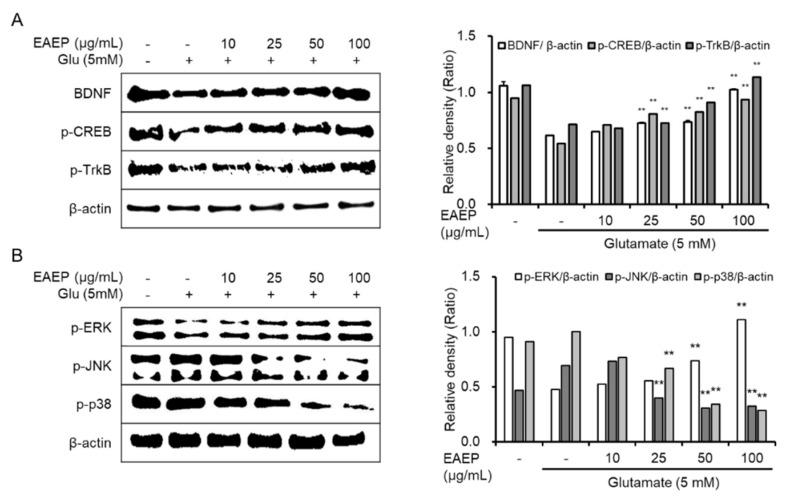
Activation of brain-derived neurotrophic factor (BDNF) expression via ERK/CREB/TrkB pathway following treatment with ethyl acetate extract of *Enteromorpha prolifera* (EAEP). (**A**) Immunoblotting and relative density of BDNF, p-CREB, p-TrkB, (**B**) p-ERK, p-JNK, and p-p38. HT-22 cells were seeded on a 60-mm dish, and then incubated for 24 h. The cells were challenged with glutamate after pre-incubation with or without EAEP (0–100 µg/mg) for 30 min. After 12 h, the expression of BDNF, p-CREB, p-TrkB, p-ERK, p-JNK, p-38 or β-actin was examined as described in the Materials and Methods. The data were pooled from three independent experiments. ** *p* < 0.01 versus glutamate-treated group. − denotes absence of EAEP.

The ERK/TrkB/CREB pathway is another mechanism underlying neuronal cell survival [33,34]. As shown in Figure 4, EAEP completely enhanced the expression of BDNF as well as p-ERK, p-CREB, and p-TrkB (Figure 4A). Based on these results, the neuroprotective effect of EAEP is closely associated with the synthesis of BDNF via ERK/CREB/TrkB pathway in neuronal cells. Since EAEP protects neuronal cell against oxidative stress via the ERK/CREB/TrkB pathway, we were interested in the intermediate protein related to mitogen-activated protein kinase (MAPK). Additionally, we confirmed the expression of JNK and p38, which belong to MAPK, in oxidative stress-induced neuronal cells. In glutamate-treated HT-22 cells, the expression of activated JNK and p38 was increased (Figure 4B). In contrast, EAEP significantly diminished their phosphorylation induced by glutamate, which was less than the control level. Therefore, we concluded that EAEP upregulated the activation of BDNF/ERK/CREB/TrkB signaling pathway and downregulated the expression of JNK and p38 in oxidative stress-induced neuronal cells, simultaneously.

### 2.5. K252a and MK2206 Inhibit the Neuroprotective Effects of EAEP

**Figure 5:**
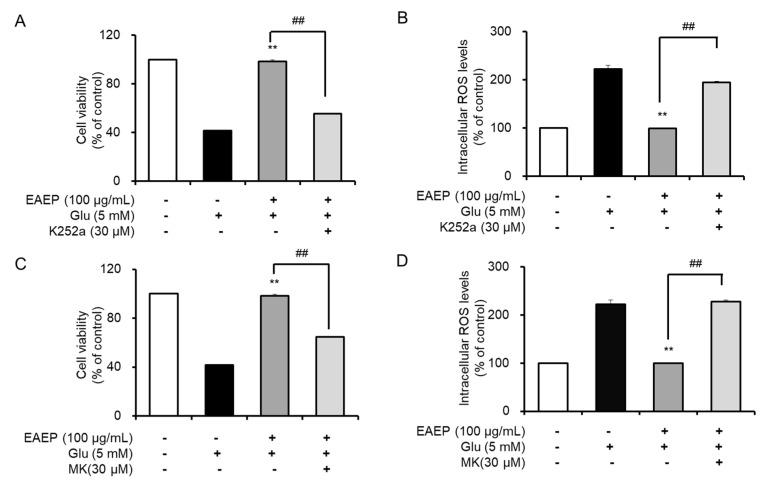
Treatment with K252a or MK-2206 inhibits neuroprotective activity of ethyl acetate extract of *Enteromorpha prolifera* (EAEP). HT-22 cells were pre-incubated with or without EAEP in combination with K252a or MK-2206 for 30 min before glutamate challenge. After 12 h, the cell viability and ROS levels were measured as described in the Materials and Methods. (**A**,**C**) Cell viability; and (**B**,**D**) ROS levels. Data represent the mean ± SEM values based on quintuple determinations. ** *p* < 0.01 versus glutamate-treated group; ^##^ *p* < 0.01 versus EAEP with glutamate-treated group. − absent, + present.

**Figure 6:**
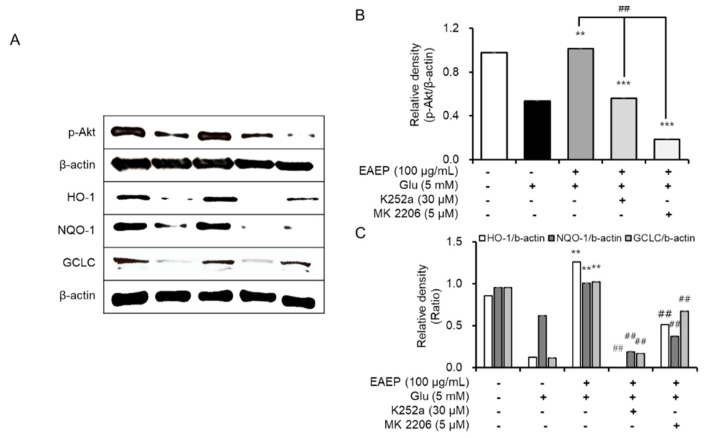
Treatment with K252a or MK-2206 inhibits neuroprotective effects of ethyl acetate extract of *Enteromorpha prolifera* (EAEP). (**A**) Immunoblotting for protein expression related with Akt/Nrf2 pathway, (**B**) relative density of p-Akt, (**C**) NQO-1, GLCL, and HO-1. HT-22 cells were pre-incubated with or without EAEP in combination with K252a or MK-2206 for 30 min before glutamate challenge. After 12 h, the expression of p-Akt, HO-1, NQO-1, GCLC, or β-actin was determined as described in the Materials and Methods. The data were obtained from three independent experiments. ** *p* < 0.01 versus glutamate-treated group; ^##^ *p* < 0.01 versus EAEP in the glutamate-treated group. − absent, + present.

**Figure 7:**
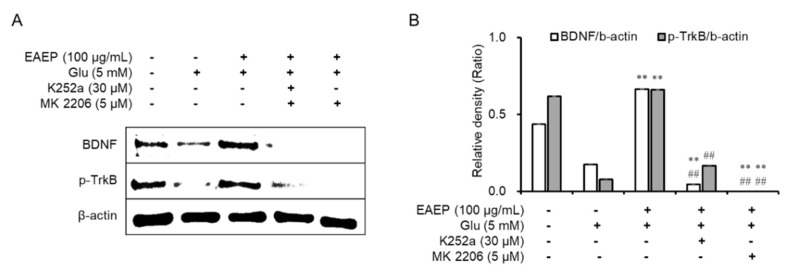
Treatment with K252a or MK-2206 inhibits neuroprotective effects of ethyl acetate extract of *Enteromorpha prolifera* (EAEP). (**A**) Immunoblotting for protein expression related with BDNF/TrkB pathway, and (**B**) relative density of BDNF and p-TrkB. HT-22 cells were pre-incubated with or without EAEP in combination with K252a or MK-2206 for 30 min before glutamate challenge. After 12 h, the expression of BDNF, p-TrkB, or β-actin was examined as described in the Materials and Methods. The data were obtained from three independent experiments. ** *p* < 0.01 versus glutamate-treated group; ^##^ *p* < 0.01 versus EAEP with glutamate-treated group. − is absence, + is present.

Subsequently, we found that the neuroprotective effects of EAEP affect both the BDNF/TrkB pathway and the Akt/Nrf2/antioxidant enzymes by treating with K252a, a TrkB inhibitor, or MK2206, a selective Akt inhibitor, We determined that the inhibitory effect dramatically neutralized the antioxidant and neuroprotective effects of EAEP in oxidative stress-induced HT-22 cells (Figure 5). Pre-treatment with either K252a or MK2206 in combination with EAEP significantly reduced the neuroprotective effect of EAEP against glutamate-treated HT-22 cells (Figure 5A,C). In addition, the inhibitor treatment interfered with the ability of EAEP to reverse ROS synthesis in oxidative stress-induced hippocampal neuronal cells (Figure 5B,D). Furthermore, both K252a and MK2006 reversed the antioxidant action of EAEP by reducing the expression of antioxidant enzymes including HO-1, NQO-1, and GCLC in oxidative stress-induced HT-22 cells (Figure 6). In particular, the expression of BDNF and p-TrkB was decreased when the inhibitors were pre-treated with EAEP (Figure 7). Collectively, these results suggest that the activation of Akt is closely associated with the BDNF/TrkB pathway as well as the expression of antioxidant enzymes in neuronal cells. Therefore, these results corroborate findings of oxidative stress prevention in hippocampal neuronal cells by EAEP by regulating the expression of BDNF and antioxidant enzymes via activation of both TrkB/BDNF and Akt/Nrf2/antioxidant enzyme pathways.

## 3. Discussion

*Enteromorpha prolifera* (EP), which is a green alga, has been used as food and traditional medicine in Eastern Asia for a long time. EP exhibits anti-diabetic, antioxidant, and anti-inflammatory activities [17,18,19,20,25,26]. Recently, the antioxidant properties of ethyl acetate extract of EP (EAEP) were attributed to the presence of diverse phytochemical ingredients, such as chlorophyll, phycocyanins, flavonoids, phenolic compounds, and polyssacharides [17,19,21,23]. In addition to phytochemicals, EAEP contains carotenoid-derived compounds associated with neuroprotective effect [12,13,14,15]. However, the neuroprotective effects of EAEP against oxidative stress-induced neurodegeneration have yet to be reported.

Recent studies suggest that the stabilization of the BDNF autocrine loop is critical for prevention and treatment of neurodegenerative diseases. Based on these findings, we hypothesized that the antioxidant property of EAEP may be related to the synthesis of both BDNF and antioxidant enzymes in oxidative stress-exposed neuronal cells [5,9,32,34]. In this study, we found that EAEP exhibits both neuroprotective and antioxidant properties mediated via BDNF expression through the ERK/CREB/TrkB pathway and antioxidant enzymes such as HO-1, NQO-1, and GCLC mediated via the Akt/Nrf2 pathway, in oxidative stress-induced neuronal cells. In contrast, EAEP attenuated the expression of JNK and p38, which belong to MAPKs along with ERK, which were reduced in oxidative stress-induced neuronal cells. Furthermore, treatment with K252a, an inhibitor of TrkB, and MK2206, a selective Akt inhibitor suppressed the neuroprotective effect of EAEP. Moreover, the expression of BDNF and antioxidant enzymes was reduced by pre-incubating the inhibitors with EAEP in oxidative stress-induced neuronal cells, which demonstrate that the activation of Akt may enhance the expression of BDNF and stabilize the activation of TrkB/Akt pathway in neuronal cells.

The neuroprotective and antioxidant mechanisms of EAEP are possibly correlated with the activation of neurotrophic signaling pathways to promote neuronal survival in neurodegenerative conditions. BDNF is the primary neurotrophic factor that enhances not only cell proliferation and growth but synaptic plasticity by activating TrkB, its receptor [7,8]. In addition, treatment with p-TrkB, an activated form of TrkB, leads to the activation of Akt, extracellular signal-regulated kinase (ERK), and cAMP-response element binding protein (CREB) pathway [5,8,31]. Recently, it was reported that the downstream phosphorylation of ERK, which is a mitogen-activated protein kinase (MAPK), activated CREB transcription, regulating the expression of BDNF [35,36]. In contrast, the phosphorylation of JNK and p38, which are also MAPKs, leads to apoptosis and cell death [37,38]. Based on these results, we suggest that ERK may promote the expression of BDNF and TrkB in oxidative stress-induced HT-22 cells.

In addition to neuronal defense system, the production of antioxidant enzymes was enhanced via activation of the TrkB/Akt/Nrf2 pathway in oxidative stress-induced neuronal cells. Glutamate toxicity is a major contributor to pathological cell death within the nervous system and appears to be mediated by reactive oxygen species [39]. High concentration of glutamate inhibits the uptake of N-acetyl cysteine, which reduces the intracellular glutathione levels and subsequent oxidative stress associated with cell death such as apoptosis and necrosis [30,31]. In particular, high concentrations of glutamate trigger oxidative glutamate toxicity in HT-22 cells, which lack functional ionotropic glutamate receptors [30,31,39]. During oxidative stress, ROS such as superoxide (O2•–) and hydrogen peroxide (H2O2) are generated mainly by mitochondria and accumulated in cells [32]. Increased ROS level is accompanied by the reduction in antioxidant enzyme expression. To suppress oxidative stress, neuronal cells activate endogenous antioxidant defense system, especially Nrf2, the key switch controlling the expression of antioxidant enzymes such as HO-1, NQO-1, and GCLC. In oxidative stress-induced neurodegenerative brains, the concentration of Nrf2 located in the cytoplasm is higher than in the nucleus and does not activate the expression of antioxidant enzymes [31]. The activation of Akt is closely linked to nuclear translocation of Nrf2 in neuronal cells [32,34]. As previously mentioned, the activation of TrkB activates Akt, which leads to nuclear translocation of cytoplasmic Nrf2. Overall, maintaining the TrkB/Akt pathway is a key strategy for the prevention and treatment of neurodegenerative diseases. The strategy is supported by evidence suggesting that EAEP reduced the formation of ROS and enhanced the phosphorylation of TrkB and Akt, and the expression of nuclear Nrf2, HO-1, NQO-1, and GCLC in oxidative stress-exposed HT-22 cells. In contrast, the addition of K252a or MK2206 attenuated the neuroprotective and antioxidant properties or EAEP by suppressing the activation of the TrkB/Akt pathway. In a previous report, we identified bioactive phytochemicals in EAEP, such as carotenoid-derived dihydroactinidiolide, canthaxanthin, fucoxanthin, violaxanthin, methionine-derived dimethylsulphoniopropionate (DMSP), chlorophyl-derived pheophorbide A, chlorophyllin, flavonoids, and phenolic compounds [23]. Based on the previous report, the neuroprotective effect of EAEP may be attributed to a synergistic effect of the bioactive compounds.

In summary, this study demonstrated that EAEP exhibits neuroprotective and antioxidant activities by promoting the synthesis of BDNF and antioxidant enzymes in oxidative stress-induced neuronal cells. The benefits of EAEP, which is rich in phytochemicals, are attributed to the stabilization of the activation of the BDNF/TrkB/Akt pathway. These results demonstrate a novel neuroprotective mechanism of EAEP against oxidative stress-induced neurodegeneration, suggesting potential role as a food supplement in the treatment and prevention of neurodegenerative diseases. Further animal studies are necessary to corroborate the neuroprotective effect of EAEP for potential clinical application.

## 4. Materials and Methods

### 4.1. Chemicals

Dulbecco’s Modified Eagle Medium (DMEM), 1× PBS and 1× Tris-buffered saline (TBS) were purchased from Welgene, Inc. (Gyeongsan, Gyeongbuk, Korea). Fetal bovine serum (FBS), 0.25% trypsin-EDTA, antibiotics, and 2,7-dichlorofluorescein diacetate (DCFDA) were obtained from Invitrogen (Carlsbad, CA, USA). K252a (a TrkB inhibitor) and MK-2206 (a specific Akt inhibitor) were purchased from Cayman Chemical Company (Ann Arbor, MI, USA). EZ-Cytox cell viability assay kit was obtained from Daeil Lab (Seoul, Korea). Specific antibodies against heme-oxygenase-1(HO-1), glutamate–cysteine ligase catalytic subunit (GCLC), and NAD(P)H quinone oxidoreductase-1 (NQO-1), NF-E2-related factor-2 (Nrf2), phosphoprotein kinase B(Akt), extracellular signal-regulated kinase (ERK), phospho-cAMP response element-binding protein (CREB), phospho-tropomyosin-related kinase receptor B(TrkB), and β-actin as well as horseradish peroxidase-conjugated IgG secondary antibodies were purchased from Cell Signaling Technology (Beverly, MA, USA). Specific antibodies against BDNF (sc-65514) were obtained from Santa Cruz Biotechnology, Inc. (Dallas, TX, USA). Muse^®^ Annexin V & Dead Cell Assay Kits were purchased from Merck Millipore, Inc. (Darmstadt, Germany). All other chemicals used in this study were analytical grade and procured from Sigma-Aldrich (St Louis, MO, USA).

### 4.2. Preparation of EAEP Extract

EAEP (Songwonfood, Seosan, South Korea) was prepared by a previous method [23]. Briefly, lyophilized EP (200 g) was extracted with 95% ethanol in a bath sonicator for 1 day, and the mixture was filtered through Whatman filter paper (No. 2). The process was repeated three times. The whole filtrate was concentrated using a rotary evaporator (Rikakikai Co. Tokyo, Japan). The concentrate was added to ethyl acetate and distilled water (1:1, *v/v*), followed by separation and evaporation of ethyl acetate layer. In a previous report, we identified a lot of phytochemicals in EAEP, including dihydroactinidiolide, canthaxanthin, fucoxanthin, violaxanthin, dimethylsulphoniopropionate (DMSP), pheophorbide A, chlorophyllin, astaxanthin, apocarotenoid, apocarotenal, chlorophyllin, lutein, zeaxanthin, flavonone, trans-stilbene, 4,4′-dinitrostilbene, triiodophloroglucinol, naringenin, phenylnaringenin, xanthone [23]. Finally, the dried residue of ethyl acetate extract (2.6 g) was dissolved in dimethyl sulfoxide (DMSO).

### 4.3. Cell Viability Assay

Cell viability was assessed according to a process reported previously [9]. HT-22 cells were pre-incubated with or without EAEP (0–100 μg/mL DMSO) for 30 min prior to glutamate treatment. After 12 h, the cell viability was assessed using an EZ-Cytox cell viability assay kit according to the manufacturer’s instructions. The absorbance at 450 nm was measured with a microplate reader (Molecular Devices, Sunnyvale, CA, USA). The percentage of surviving cells was determined relative to the control values.

### 4.4. Measurement of Intracellular ROS Level

The level of intracellular ROS was measured using 2′,7′-dichlorofluorescein diacetate (DCFDA) following a previous method [9]. After glutamate treatment for 7 h, the cells were stained with 10 μM DCFDA in Hank’s balanced salt solution (HBSS) for 30 min in darkness. The plate was measured by microplate reader (Beckman Coulter DTX 880 Multimode Detector, Brea, CA, USA) at an excitation wavelength of 485 nm and an emission wavelength of 525 nm.

### 4.5. Flow Cytometry Analysis

To measure apoptotic bodies, HT-22 cells were seeded. After glutamate treatment for 12 h, all the cells were harvested. Dead cells including both apoptotic cells and necrotic cells were measured by using Muse Annexin V & Dead Cell Assay kit following the manufacturer’s instruction. Finally, stained cells were analyzed by a flow cytometer (Muse^TM^ Cell Analyzer, Merck Millipore, Darmstadt, Germany) with Muse 1.1.2 analysis software.

### 4.6. Protein Determination

Proteins were measured with a Bio-Rad protein assay dye reagent [40]. The cell lysate mixed with a 1:20 diluted dye reagent was incubated at room temperature for 10 min. The mixture was measured with a spectrophotometer at 595 nm. Bovine serum albumin (BSA) was used to obtain the standard curve in the range of 0.2–1.0 mg/mL.

### 4.7. Extraction of Nuclear and Cytosolic Protein

Nuclear and cytosolic proteins were fractionated using a Nuclear Extraction Kit (Cayman Chemicals, Ann Arbor, MI) according to the manufacturer’s instructions. Briefly, cells were harvested and centrifuged (3000 rpm, 5 min) at 4 °C. The cell pellets were mixed with hypotonic buffer containing a phosphatase inhibitor and a protease inhibitor. After 10 min of incubation on ice, the cells were treated with 10% Nonidet P40 Assay Reagent. Nuclei were recovered by centrifugation (14,000 rpm, 30 s), and the supernatant was stored as a cytoplasmic extract at −80°C until use. The nuclei were extracted with Nuclear Extraction Buffer for 30 min on ice. Insoluble material was removed by centrifugation (14,000 rpm, 10 min). Finally, the supernatant was used as a nuclear extract.

### 4.8. Western Blot Analysis

Western blot analysis was used to determine the protein expression of HO-1, NQO-1, GCLC, Nrf2, p-Akt, p-TrkB, BDNF, p-ERK, p-CREB, Lamin B, and β-acitn in HT-22 cells. Briefly, cellular proteins were extracted using the PRO-PREP Protein Extraction solution (iNtRON Biotechnology, Gyeonggi-do, Korea) according to the manufacturer’s instructions. The proteins (20–60 μg) obtained from the supernatant were resolved by SDS-PAGE, and transferred onto a polyvinylidene fluoride (PVDF) membrane. The nonspecific binding of antibodies was blocked using 5% BSA in TBS buffer (20 mM Tris-HCl + 150 mM NaCl, pH 7.4) for 2 h, and the membranes were probed with different primary antibodies. The membranes were incubated with horseradish peroxidase-conjugated anti-mouse IgG or anti-rabbit IgG for 1 h for the immunoblotting analysis, followed by visualization using the WEST OneTM western blot detection system (iNtRON Biotechnology, Inc, Gyeonggi-do, Korea). The relative density of the protein expression was quantitated by densitometry (Image J, National Institutes of Health, Bethesda, Maryland, USA).

### 4.9. Statistical Analysis

All the results were expressed as the mean ± SEM. The statistical analysis was performed using SPSS 24.0 program (SPSS Inc., Chicago, IL, USA). Data were analyzed by one-way analysis of variance (ANOVA) followed by an LSD test and post-hoc comparison with Duncan’s multiple-range test. Statistical significance was considered at * *p* < 0.05, ** *p* < 0.01, or *** *p* < 0.001 for the comparison involving only glutamate-treated HT-22 cells. The statistical significance was considered at ^#^ *p* < 0.05, ^##^ *p* < 0.01, or ^###^ *p* < 0.001 for comparison with glutamate-treated HT-22 cells exposed to EAEP 100 μg/mL.

## 5. Conclusions

The present study demonstrated that EAEP exerts neuroprotective action by protecting against glutamate-induced apoptosis via the TrkB/Akt signaling pathway in oxidative stress-induced hippocampal neuronal cells. Our study suggests that the neuroprotective effect of EAEP may provide further information for the application of EAEP as a candidate for the prevention and treatment of neurodegenerative disorders.

## Abbreviations

Akt	protein kinase B
BDNF	brain-derived neurotrophic factor
CREB	cAMP response element-binding protein
EAEP	ethyl acetate with *Enteromorpha prolifera*
GCLC	glutamate–cysteine ligase catalytic subunit
HO-1	heme oxygenase-1
NQO-1	NAD(P)H quinine oxidoreductase-1
Nrf2	NF-E2-related factor-2
ROS	reactive oxygen species
TrkB	Tropomyosin-related kinase receptor B
